# A Fault-Tolerant Polar Grid SINS/DVL/USBL Integrated Navigation Algorithm Based on the Centralized Filter and Relative Position Measurement

**DOI:** 10.3390/s19183899

**Published:** 2019-09-10

**Authors:** Lin Zhao, Yingyao Kang, Jianhua Cheng, Mouyan Wu

**Affiliations:** College of Automation, Harbin Engineering University, Harbin 150001, China

**Keywords:** polar navigation, grid strapdown inertial navigation system (SINS)/Doppler velocity log (DVL)/ultra-short baseline (USBL) integrated navigation, relative position measurement, vector fault detection method

## Abstract

Navigation is a precondition for ocean space vehicles to work safely in polar regions. The traditional polar algorithms employ the grid strapdown inertial navigation system (SINS) as the backbone and Doppler velocity log (DVL) output velocity as measurements to constitute the integrated navigation system, of which, however, the position errors still accumulate with time. The ultra-short baseline (USBL) position system can provide position information that can be used to improve the performance of the SINS/DVL integrated system. Therefore, a grid SINS/DVL/USBL integrated algorithm for polar navigation is proposed in this paper. In order to extend the availability of the USBL and improve integration accuracy in polar regions, the USBL observation model is established based on the relative position measurement firstly. Then, a grid SINS/DVL/USBL integrated algorithm is proposed to fuse the information of these sensors with a modified Kalman filter (MKF) dealing with the sparse USBL output. Finally, a vector fault detection method, which takes the measurements as detection objects instead of the filter, is designed to locate the measurement fault and can be employed by the centralized filter to improve the fault-tolerant. Simulation and experiment results show that the proposed grid SINS/DVL/USBL integrated navigation system can further restrain SINS errors especially the position errors effectively. Meanwhile, the vector fault detection method can detect and isolate the fault measurements of centralized filter immediately and accurately. Therefore, the proposed fault-tolerant grid SINS/DVL/USBL integrated navigation algorithm can improve the reliability and accuracy of polar navigation for ocean space application.

## 1. Introduction

More and more significant scientific research is carried out in the ocean of the polar regions [1,2]. The high-precision and highly reliable navigation is the precondition for ocean space vehicles to operate normally and safely [3]. The strapdown inertial navigation system (SINS) has been widely used for polar vehicles, especially for the underwater vehicles due to its highly autonomous [4]. However, the SINS output contains periodic oscillation errors and accumulated errors. Navigation with a single sensor or a single system is often insufficient, so the SINS-based integrated navigation technology is a potential method for polar navigation [5,6,7,8]. 

The traditional integrated navigation algorithms are based on a north-oriented geographic framework and will lose efficacy in polar regions because of the meridian convergence [9]. A grid frame and the grid SINS algorithm have been proposed in [10], which are employed in this paper to solve the problem of meridian convergence. In polar regions, the geomagnetic navigations may fail to work due to the anomaly of geomagnetic field. The visual navigation has a high demand for visibility. The geophysical field navigation has strong dependence on the completeness of the database. The global navigation satellite system (GNSS) can provide the position or some other information to construct the grid SINS/GNSS integration, but cannot provide continuous information for underwater vehicles, which may decrease the usability and accuracy of the grid SINS/GNSS integration [11]. The acoustic sensors have high accuracy for ocean space applications and will not lose performance in polar regions [12], so they can provide external measurements to restrain the SINS errors [13,14,15]. However, the reliability of acoustic sensors is influenced by the operation environment. In this paper, an integrated navigation algorithm consisting of the grid SINS and acoustic sensors is proposed with a novel fault detection method to achieve a high-precision and high-reliability polar navigation performance.

It is known that the Doppler velocity log (DVL) is widely used to provide velocities [16], and the grid SINS/DVL integrated algorithm has been proposed in [17]. But the position errors of a grid SINS/DVL integrated algorithm still accumulate with time. To overcome this problem, some additional position measurements could be introduced. The majority of traditional integrated systems choose the absolute position as measurements [18], but in polar regions the longitude errors will be amplified and no longitude can describe the position of the North or South Pole [10]. The ultra-short base line (USBL) position system can provide position information for both the surface and underwater vehicles. The output relative positions are provide as original information [12,19], i.e., the range *R*, altitude angle α and azimuth angle β between the transponder and the vehicle-mounted hydrophone array, and then the absolute positions, i.e., the position coordinate in the earth-centered earth-fixed (ECEF) frame, can be calculated based on the relative positions [20]. Influenced by the operation environment, the USBL can only provide one kind relative position in some cases, such as only the angle α and β, so the absolute position cannot be provided in these cases. Therefore, the original relative position has more availability than the absolute position. If the relative position is chosen as the measurements, together with a proper fault detection method to detect the effective measurements, the availability of the USBL can be extended, and the integrated filter will have more accuracy and stability than the filter with absolute position measurements. A grid-based USBL filter observation model can be designed with the relative position measurements, and then the grid SINS/DVL/USBL filter can be proposed to improve the navigation accuracy.

The acoustic sensors may be failed due to the operation environment [21], and with the increase of the measurement information, the fault occurrence may also increase. Although some robust Kalman filtering approaches have been studied with uncertainties such as model validation-based robust Kalman filtering with uncertainties satisfying integral quadratic constraints [22], interval Kalman filtering [23], and robust Kalman filtering for systems with stochastic uncertainties [24], which can improve the robustness of the system despite the model uncertainties or even the linearization errors, these methods still cannot detect and exclude the fault measurements. In addition, the output of the USBL is one kind of sparse measurement signals [25] and influenced by the application environment, the acoustic sensors may have an unstable update frequency, which also cannot be solved by these robust Kalman filtering approaches. Therefore, a fault detection method is essential to deal with the measurement failure firstly. Then, a new or modified Kalman filter (MKF) algorithm to deal with the measurements with low or unstable frequency is required. 

The wavelet filter, sequential probability ratio test and residual $\chi^{2}$ are traditional methods to detect the measurement fault for the integration filter [26]. But the traditional detection methods are scalar detection methods, which virtually take the filter as object to detect and locate the fault filter instead of the measurement. Therefore, the traditional scalar methods can only be employed by the sub-optimal federal filter to detect and isolate the sub-filter fault, but cannot be employed by the centralized filter. What is more, the scalar methods also take the filter as object to isolate the fault, which may be a waste of useful signals because the isolated filter may contain some other fault-free measurements. For the polar underwater vehicles, the reference navigation information is not as abundant as in the low and middle latitude regions, and the effective measurements should be utilized fully to obtain a proper navigation performance. Therefore, in order to obtain proper accuracy and maintain high reliability, the centralized filters together with a novel detection method are more proper choice for polar vehicles than the federal filter with traditional scalar detection methods. The novel detection method should take the measurement as object to deal with measurement fault and improve the utilization of effective measurements. 

In this paper, a fault-tolerant grid SINS/DVL/USBL integrated navigation algorithm is proposed to improve the navigation reliability and accuracy in polar regions for ocean space application. The DVL and USBL outputs are introduced as measurements, and the USBL observation model is designed based on the grid frame with relative positions as measurements to overcome the position task. Then the grid SINS/DVL/USBL centralized filter model is proposed for polar navigation. Besides, to deal with the sparse USBL output measurements, an MKF is employed to maintain the high and stable filter update frequency and accuracy. Finally, a vector fault detection method is designed to improve the fault-tolerant of integration. In the vector method, the fault detection vector is designed based on the classified measurements. Each element of the fault detection vector represents the quality of the corresponding measurement group. Therefore, the measurement is taken as the object to be detected and isolated instead of the filter, which can improve the utilization of effective measurements and fault-tolerant of the filter, especially the centralized filter. All of the design in the above will improve the accuracy and reliability of the integrated navigation system for vehicles in polar regions, and some experiments are conducted to validate the performance of the proposed integrated navigation algorithm.

The remainder sections are organized as follows. Section 2 describes the grid SINS and USBL position system. The grid SINS/DVL/USBL integrated filter model is proposed in Section 3. Section 4 explores the MKF filter algorithm and vector fault detection method. Simulations and semi-physical experiments are conducted in Section 5 to validate the performance of the proposed integrated navigation algorithm. Finally, Section 6 outlines the conclusions and future works.

## 2. The Grid SINS and USBL Position System 

The grid SINS, which is chosen as a backbone navigation system, is employed with the relative position from the USBL and velocity from the DVL to compose an integrated system for the polar navigation. Besides, some different frames are reviewed here, i.e., the inertial frame i, the earth centred earth fixed frame ECEF(e), the geographic frame g, the grid frame G, the body frame b, and the USBL array frame u.

### 2.1. The Grid Frame and Grid SINS Mechanization

**Figure 1:**
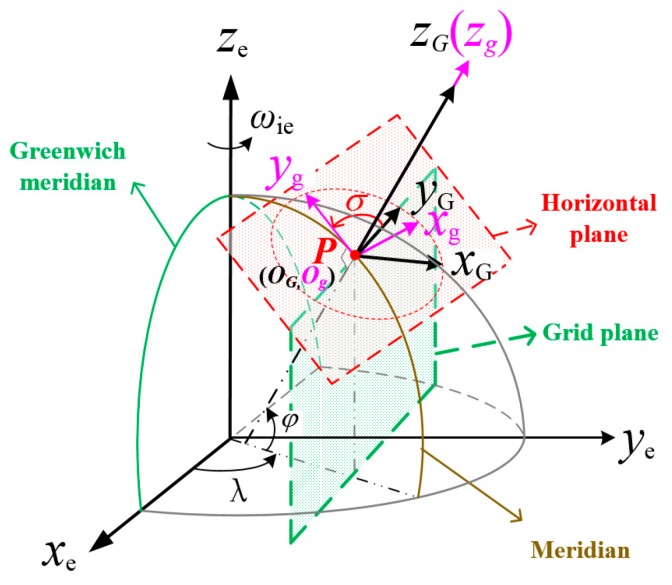
Description of the grid frame.

As shown in Figure 1, the grid SINS chooses the right-handed grid frame as the navigation frame. 

A grid north axis ($y_{G}$ - axis), which is parallel to the Greenwich plane, is employed instead of the geographic north, and the angle between the grid north and geographic north is σ. The other detail descriptions of the grid frame can be found in [10].

The mechanization of grid SINS attitude, velocity, and position are:

(1) $${\dot{C}}_{b}^{G}=C_{b}^{G}(\omega_{Gb}^{G}\times )$$

(2) $${\dot{V}}^{G}=C_{b}^{G}f^{b}-\left(2\omega_{ie}^{G}+\omega_{eG}^{G}\right)\times V^{G}+g^{G}$$

(3) $${\dot{R}}^{e}=C_{G}^{e}V^{G}=(C_{e}^{g}){}^{T}{\left(C_{g}^{G}\right)}^{T}V^{G}$$

The detailed processing and physical variables of (1), (2) and (3) can refer to [10].

### 2.2. The USBL Position System

**Figure 2:**
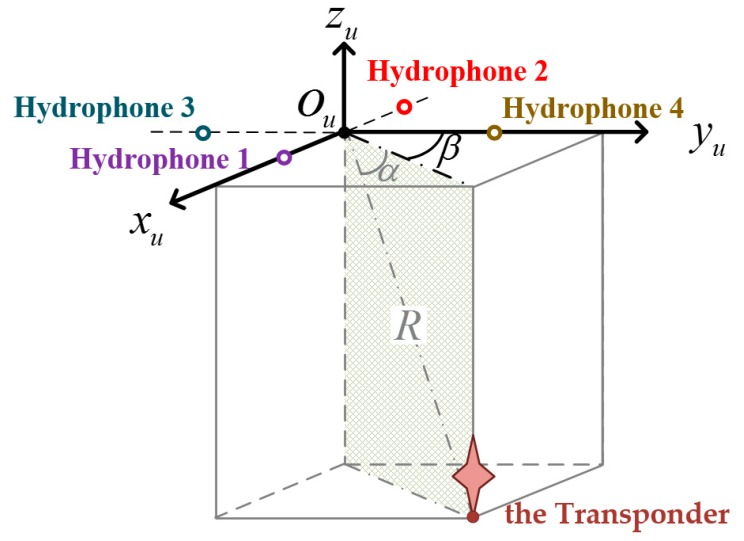
Description of the ultra-short baseline (USBL) array frame and relative position.

As shown in Figure 2, the USBL position system consists of four vehicle-mounted hydrophones and a known-location transponder (denoted as r). The four hydrophones are arranged in two orthogonal baselines and make up the USBL array. The intersection point of two baselines is the origin of the USBL array frame (u frame) and the right-handed u frame are then shown as follows: 

It is assumed that the sound velocity and wavelength under water are known. Then, the sound signals transmit between the USBL array and the transponder to measurement the relative position between them. As shown in Figure 2, the relative position between the USBL array and the transponder can be presented as $\left[\begin{array}{ccc} \alpha & \beta & R \end{array}\right]$, where α s the altitude angle, and β is the azimuth angle, and R is the range.

## 3. Design of the Grid SINS/DVL/USBL Integrated Filter Model with Relative Position Measurements

**Figure 3:**
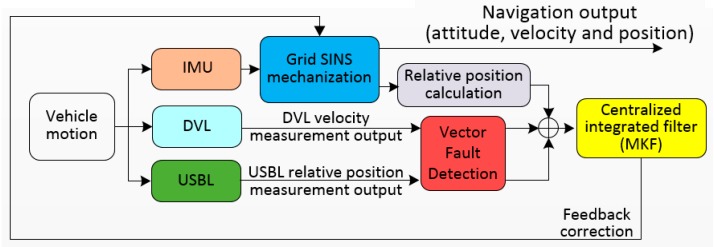
Diagram of the proposed grid SINS/DVL/USBL integrated navigation algorithm.

As discussed above, the single navigation device cannot maintain the accuracy of polar navigation. Therefore, a polar grid SINS/DVL/USBL integrated navigation algorithm is proposed to enhance the navigation performance for vehicles. In this section, the grid SINS/DVL/USBL filter model is designed for the proposed integrated algorithm. The DVL provides the absolute velocity while the USBL provides the relative position $\left[\begin{array}{ccc} \alpha & \beta & R \end{array}\right]$ as the external navigation information. Figure 3 shows the diagram of the proposed grid SINS/DVL/USBL integrated navigation algorithm. The design details of the algorithm will be discussed in the following subsections and Section 4.

### 3.1. Design of the Dynamic Models

The dynamic models of the integrated navigation system include the grid SINS, DVL and USBL models, which can be described by state equations as follows:

(4) $$\dot{X}=FX+GW$$

where the filter state is $X={\left[\begin{array}{ccc} x_{\mathrm{SIN}S} & x_{\mathrm{DVL}} & x_{\mathrm{USBL}} \end{array}\right]}^{T}$; the filter state noise is $W={\left[\begin{array}{ccc} w_{\mathrm{SIN}S} & w_{\mathrm{DVL}} & w_{\mathrm{USBL}} \end{array}\right]}^{T}$; F is the system matrix and $F=\left[\begin{array}{ccc} F_{SINS} & 0_{15\times 3} & 0_{15\times 6}\\ 0_{3\times 15} & F_{DVL} & 0_{3\times 6}\\ 0_{6\times 15} & 0_{6\times 3} & F_{USBL} \end{array}\right]$; G is the noise transition matrix and $G=\left[\begin{array}{ccc} G_{SINS} & 0_{15\times 3} & 0_{15\times 2}\\ 0_{3\times 6} & G_{DVL} & 0_{3\times 2}\\ 0_{6\times 6} & 0_{6\times 3} & G_{USBL} \end{array}\right]$. $x_{\mathrm{SINS}}$, $x_{\mathrm{DVL}}$ and $x_{\mathrm{USBL}}$ are the system states of SINS, DVL and USBL respectively. $w_{\mathrm{SINS}}$, $w_{\mathrm{DVL}}$ and $w_{\mathrm{USBL}}$ are the system noises of SINS, DVL and USBL respectively. $F_{SINS}$, $F_{DVL}$ and $F_{USBL}$ are the system matrixes of SINS, DVL and USBL respectively. $G_{SINS}$, $G_{DVL}$ and $G_{USBL}$ are the noise transition matrixes of SINS, DVL and USBL respectively. 

The detail of the dynamic models will be described in the follow sub-sections, where the dynamic model of the USBL based on relative position measurements will be discussed in particular. 

#### 3.1.1. Dynamic Model of the Grid SINS and DVL

According to [17], the state of grid SINS to be estimated is chosen as $x_{SINS}={\left[\begin{array}{ccccc} \phi & \delta V^{G} & \delta R^{e} & \varepsilon^{b} & \nabla^{b} \end{array}\right]}^{T}$ and the differential equations of grid SINS state can be described as:

(5) $$\{\begin{array}{l} \dot{\phi }=-\omega_{iG}^{G}\times \phi +C_{V\_\phi }\delta V+C_{R\_\phi }\delta R^{e}-C_{b}^{G}\varepsilon^{b}\\ \delta {\dot{V}}^{G}=f^{G}\times \phi +C_{V\_V}\delta V^{G}+C_{R\_V}\delta R^{e}+C_{b}^{G}\nabla^{b}\\ \begin{array}{l} \delta {\dot{R}}^{e}=C_{V\_R}\delta V^{G}+C_{R\_R}\delta R^{e}\\ {\dot{\varepsilon }}^{b}=0 \end{array}\\ {\dot{\nabla }}^{b}=0 \end{array}$$

where $\varepsilon^{b}$ is the gyroscope drift, $\nabla^{b}$ is the accelerometer bias, ϕ is the attitude error, $\delta V^{G}$ is the velocity error, and $\delta R^{e}$ is the position error in ECEF frame. The $C_{V\_\phi }$, $C_{R\_\phi }$, $C_{V\_V}$, $C_{R\_V}$, $C_{V\_R}$, and $C_{R\_R}$ are the sub-state transition matrixes, which can refer to [17].

Besides, the DVL state is random velocity error, i.e., $x_{DVL}=\delta V_{DVL}^{m}$, which is assumed as the one-order Markov process:

(6) $$\delta {\dot{V}}_{DVL}^{m}=-\delta V_{DVL}^{m}/\tau_{V}+w_{V}$$

where $\tau_{V}$ is the correlation time of the Markov process and $w_{V}$ is the zero-mean Gaussian white noise.

Therefore, the grid SINS and DVL dynamic models can be described as (7) and (8), respectively.

(7) $${\dot{x}}_{\mathrm{SINS}}=F_{SINS}x_{SINS}+G_{SINS}w_{SINS}$$

(8) $${\dot{x}}_{\mathrm{DVL}}=F_{\mathrm{DVL}}x_{\mathrm{DVL}}+G_{\mathrm{DVL}}w_{\mathrm{DVL}}$$

#### 3.1.2. Dynamic Model of the USBL Based on Relative Position Measurements

The USBL output direction errors ${\left[\begin{array}{cc} \delta \alpha_{U} & \delta \beta_{U} \end{array}\right]}^{T}$, the range factor error $\delta K_{U}$, and the USBL installation error $\psi_{U}$ are chosen as the USBL state, and it can be expressed as $x_{\mathrm{USBL}}={\left[\begin{array}{cccc} \delta \alpha_{U} & \delta \beta_{U} & \delta K_{U} & \psi_{U} \end{array}\right]}^{T}$. 

The range R between the transponder and USBL array can be obtained as:

(9) $$R=\frac{cT}{2}$$

where c is the sound velocity. T is the signal’s roundtrip time between the transponder and USBL array.

Considering of the range error δR, the range R can be rewritten as:

(10) $$R+\delta R=(1+\delta K_{u})R$$

where $\delta K_{U}$ is the range factor error, which is related to the scale factor error.

After the calibration, the USBL installation error $\psi_{U}$ and the range factor error $\delta K_{U}$ are considered as the random constants. The direction measurement errors ${\left[\begin{array}{cc} \delta \alpha_{U} & \delta \beta_{U} \end{array}\right]}^{T}$ are considered as the one-order Markov processes:

(11) $$\left\{ \begin{array}{l} \delta {\dot{\alpha }}_{U}=-\delta \alpha_{U}/\tau_{\alpha }+w_{\alpha }\\ \delta {\dot{\beta }}_{U}=-\delta \beta_{U}/\tau_{\beta }+w_{\beta } \end{array}\right.$$

where τ is the correlation time of Markov process and w is the zero-mean Gaussian white noise.

The USBL dynamic model is described as:

(12) $${\dot{x}}_{\mathrm{USBL}}=F_{\mathrm{USBL}}x_{\mathrm{USBL}}+G_{\mathrm{USBL}}w_{\mathrm{USBL}}$$

where $F_{USBL}$ is the system matrix and $F_{USBL}=\mathrm{diag}(\begin{array}{cccccc} -1/\tau_{\alpha } & -1/\tau_{\beta } & 0 & 0 & 0 & 0 \end{array})$; $G_{USBL}$ is the noise transition matrix and $G_{USBL}={\left[\begin{array}{cc} I_{2\times 2} & 0_{4\times 2} \end{array}\right]}^{T}$.

### 3.2. Design of the Observation Models

The observation models of the grid SINS/DVL/USBL integrated navigation system include the DVL model and the USBL model, which can be described as follows:

(13) Z=HX+V

where $Z={\left[\begin{array}{cc} z_{\mathrm{DVL}} & z_{\mathrm{USBL}} \end{array}\right]}^{T}$ is the filter observation, $z_{\mathrm{DVL}}$ and $z_{\mathrm{USBL}}$ are the observations of the DVL and USBL, respectively; $H={\left[\begin{array}{cc} H_{DVL} & H_{USBL} \end{array}\right]}^{T}$ is the observation matrix, $H_{DVL}$ and $H_{USBL}$ are the observation matrixes of the DVL and USBL, respectively; $V={\left[\begin{array}{cc} v_{\mathrm{DVL}} & v_{\mathrm{USBL}} \end{array}\right]}^{T}$ is the observation noise, $v_{\mathrm{DVL}}$ and $v_{\mathrm{USBL}}$ are the observation noises of the DVL and USBL, respectively.

The detail of observation models will be described in the following sub-sections where the observation model of the USBL based on relative position measurements will be discussed in particular. 

#### 3.2.1. Observation Model of the DVL

Both the grid SINS and DVL can output vehicle velocity, which is chosen as one of the system observations.

After calibration, the DVL installation error and scale factor error are compensated and neglected in this paper. The DVL output in *G* frame can be obtained as follows [17]:

(14) $${\tilde{V}}_{DVL}^{G}=V+V_{DVL}^{G}(V_{DVL}^{G}\times )\varphi +\delta V_{DVL}^{G}$$

where $V^{G}$ is the actual velocity and $\delta V_{DVL}^{G}$ is the random velocity error of DVL in the G frame.

Besides, the grid SINS output velocity can be described as:

(15) $${\tilde{V}}_{SINS}^{G}=V^{G}+\delta V_{SINS}^{G}$$

where $\delta V_{SINS}^{G}$ is the SINS velocity error in G frame.

Therefore, the DVL observation model of the integrated system can be given as:

(16) $$Z_{DVL}={\tilde{V}}_{SINS}^{G}-{\tilde{V}}_{DVL}^{G}=H_{DVL}x+v_{DVL}$$

where $H_{DVL}$ can be described as: $H_{DVL}=\left[\begin{array}{ccc} -V_{DVL}^{G}\times & I_{3\times 3} & 0_{3\times 18} \end{array}\right]$.

#### 3.2.2. Observation Model of the USBL Based on Relative Position Measurements

The USBL position system can provide the direction and range between the USBL array and transponder expressed as $\left[\begin{array}{ccc} \alpha_{U} & \beta_{U} & R_{U} \end{array}\right]$. The direction and range between USBL array and transponder can also be calculated by the grid SINS output and expressed as $\left[\begin{array}{ccc} \alpha_{S} & \beta_{S} & R_{S} \end{array}\right]$. Then the USBL observation is expressed as:

(17) $$Z=\left[\begin{array}{c} \alpha_{U}-\alpha_{S}\\ \beta_{U}-\beta_{S}\\ R_{U}-R_{S} \end{array}\right]=\left[\begin{array}{c} \alpha +{\delta \alpha }_{U}\\ \beta +{\delta \beta }_{U}\\ R+{\delta R}_{U} \end{array}\right]-\left[\begin{array}{c} \alpha +{\delta \alpha }_{S}\\ \beta +{\delta \beta }_{S}\\ R+{\delta R}_{S} \end{array}\right]=\left[\begin{array}{c} {\delta \alpha }_{U}\\ {\delta \beta }_{U}\\ {\delta R}_{U} \end{array}\right]-\left[\begin{array}{c} {\delta \alpha }_{S}\\ {\delta \beta }_{S}\\ {\delta R}_{S} \end{array}\right]$$

where the $\left[\begin{array}{ccc} {\delta \alpha }_{U} & {\delta \beta }_{U} & {\delta R}_{U} \end{array}\right]$ and $\left[\begin{array}{ccc} {\delta \alpha }_{S} & {\delta \beta }_{S} & {\delta R}_{S} \end{array}\right]$ are relative position errors of the USBL output and grid SINS output, respectively. 

The $\left[\begin{array}{ccc} {\delta \alpha }_{U} & {\delta \beta }_{U} & {\delta R}_{U} \end{array}\right]$ can be described as:

(18) $$\left[\begin{array}{c} \delta \alpha_{U}\\ \delta \beta_{U}\\ \delta R_{U} \end{array}\right]=\left[\begin{array}{cc} I_{2\times 2} & 0_{2\times 1}\\ 0_{1\times 2} & R \end{array}\right]\left[\begin{array}{c} \delta \alpha_{U}\\ \delta \beta_{U}\\ \delta K_{U} \end{array}\right]=H_{U}\left[\begin{array}{c} \delta \alpha_{U}\\ \delta \beta_{U}\\ \delta K_{U} \end{array}\right]$$

The position coordinate of transponder *r* relative to USBL array in *u* frame can be calculated and obtained from the grid SINS output, which is defined as $P_{ur}^{u}{}_{S}={\left[\begin{array}{ccc} x_{S}^{u} & y_{S}^{u} & z_{S}^{u} \end{array}\right]}^{T}$. $P_{ur}^{u}{}_{S}$ can be obtained by:

(19) $$P_{ur}^{u}{}_{S}=C_{b}^{u}C_{G}^{b}C_{g}^{G}C_{e}^{g}(P_{r}^{e}-P_{b}^{e}{}_{S}-C_{G}^{e}C_{b}^{G}\delta P_{bu}^{b})={\left[\begin{array}{ccc} x_{S}^{u} & y_{S}^{u} & z_{S}^{u} \end{array}\right]}^{T}$$

where $P_{r}^{e}$ is the position of the transponder expressed in *e* frame as a known quantity. $P_{b}^{e}{}_{S}$ is the position of *b* frame expressed in *e* frame and provided by the grid SINS. $C_{b}^{u}$ and $\delta P_{bu}^{b}$ are the calibration matrix and the level arm between the b frame and u frame and obtained by off-line calibrations. $\left[\begin{array}{ccc} \alpha_{S} & \beta_{S} & R_{S} \end{array}\right]$ can be obtained by:

(20) $$\left[\begin{array}{c} \alpha_{S}\\ \beta_{S}\\ R_{S} \end{array}\right]=\left[\begin{array}{c} \arctan\left(z_{S}^{u}/\sqrt{{\left(y_{S}^{u}\right)}^{2}+{\left(x_{S}^{u}\right)}^{2}}\right)\\ \arctan\left(x_{S}^{u}/y_{S}^{u}\right)\\ \sqrt{{\left(x_{S}^{u}\right)}^{2}+{\left(y_{S}^{u}\right)}^{2}+{\left(z_{S}^{u}\right)}^{2}} \end{array}\right]$$

The first order total differential of (20) is written as:

(21) $$\begin{array}{ll} {\left[\begin{array}{ccc} {\delta \alpha }_{S} & {\delta \beta }_{S} & {\delta R}_{S} \end{array}\right]}^{T} & =\left[\begin{array}{ccc} \frac{-x_{S}^{u}z_{S}^{u}}{R_{S}{}^{2}\sqrt{{\left(x_{S}^{u}\right)}^{2}+{\left(y_{S}^{u}\right)}^{2}}} & \frac{-x_{S}^{u}y_{S}^{u}}{R_{S}{}^{2}\sqrt{{\left(x_{S}^{u}\right)}^{2}+{\left(y_{S}^{u}\right)}^{2}}} & \frac{\sqrt{{\left(x_{S}^{u}\right)}^{2}+{\left(y_{S}^{u}\right)}^{2}}}{R_{S}{}^{2}}\\ \frac{y_{S}^{u}}{{\left(x_{S}^{u}\right)}^{2}+{\left(y_{S}^{u}\right)}^{2}} & \frac{-x_{S}^{u}}{{\left(x_{S}^{u}\right)}^{2}+{\left(y_{S}^{u}\right)}^{2}} & 0\\ \frac{x_{S}^{u}}{R_{S}} & \frac{y_{S}^{u}}{R_{S}} & \frac{z_{S}^{u}}{R_{S}} \end{array}\right]\left[\begin{array}{c} {\delta x}_{S}^{u}\\ {\delta y}_{S}^{u}\\ {\delta z}_{S}^{u} \end{array}\right]\\ & =A{\left[\begin{array}{ccc} {\delta x}_{S}^{u} & {\delta y}_{S}^{u} & {\delta z}_{S}^{u} \end{array}\right]}^{T} \end{array}$$

The error of $P_{ur}^{u}{}_{S}$ is denoted as $\delta P_{ur}^{u}{}_{S}={\left[\begin{array}{ccc} \delta x_{S}^{u} & \delta y_{S}^{u} & \delta z_{S}^{u} \end{array}\right]}^{T}$. Considering of $\delta P_{ur}^{u}{}_{S}$, equation (19) can be rewritten as:

(22) $$\begin{array}{l} {\left[\begin{array}{ccc} x_{S}^{u}+\delta x_{S}^{u} & y_{S}^{u}+\delta y_{S}^{u} & z_{S}^{u}+\delta z_{S}^{u} \end{array}\right]}^{T}\\ =C_{b}^{u}(I-\psi_{U}\times )C_{G}^{b}(I+\phi \times )(C_{e}^{G}+\delta C_{e}^{G})(P_{br}^{e}+\delta P_{br}^{e})-C_{b}^{u}(I-\psi_{U}\times )P_{bu}^{b}\\ =P_{ur}^{u}{}_{S}-C_{b}^{u}P_{bu}^{b}-C_{b}^{u}(\psi_{U}\times )C_{G}^{b}C_{e}^{G}P_{br}^{e}\\ +C_{b}^{u}(\psi_{U}\times )P_{bu}^{b}+C_{b}^{u}C_{G}^{b}(\phi \times )C_{e}^{G}P_{br}^{e}+C_{b}^{u}C_{G}^{b}\delta C_{e}^{G}P_{br}^{e}+C_{b}^{u}C_{G}^{b}C_{e}^{G}\delta P_{br}^{e} \end{array}$$

Then $\delta P_{ur}^{u}{}_{S}$ can be expressed as:

(23) $$\begin{array}{l} {\left[\begin{array}{lll} \delta x_{S}^{u} & \delta y_{S}^{u} & \delta z_{S}^{u} \end{array}\right]}^{T}\\ =-C_{b}^{u}(\psi_{U}\times )C_{G}^{b}C_{e}^{G}P_{br}^{e}+C_{b}^{u}(\psi_{U}\times )P_{bu}^{b}+C_{b}^{u}C_{G}^{b}(\phi \times )C_{e}^{G}P_{br}^{e}+C_{b}^{u}C_{G}^{b}\delta C_{e}^{G}P_{br}^{e}+C_{b}^{u}C_{G}^{b}C_{e}^{G}\delta P_{br}^{e}\\ =-C_{b}^{u}C_{G}^{b}(C_{e}^{G}P_{br}^{e}\times )\phi +[C_{b}^{u}C_{G}^{b}[(C_{e}^{G}P_{br}^{e})\times ]C_{R2\delta \theta }-C_{b}^{u}C_{G}^{b}C_{e}^{G}]\delta R^{e}+[C_{b}^{u}(C_{G}^{b}C_{e}^{G}P_{br}^{e}\times )-C_{b}^{u}(P_{bu}^{b}\times )]\psi_{U} \end{array}$$

where $C_{R2\delta \theta }$ can refer to [17], and equation (21) can be rewritten as:

(24) $$\begin{array}{ll} {\left[\begin{array}{lll} {\delta \alpha }_{S} & {\delta \beta }_{S} & {\delta R}_{S} \end{array}\right]}^{T} & =A{\left[\begin{array}{lll} {\delta x}_{S}^{u} & {\delta y}_{S}^{u} & {\delta z}_{S}^{u} \end{array}\right]}^{T}\\ & =-AC_{b}^{u}C_{G}^{b}(C_{e}^{G}P_{br}^{e}\times )\phi +A[C_{b}^{u}C_{G}^{b}[(C_{e}^{G}P_{br}^{e})\times ]C_{R2\delta \theta }-C_{b}^{u}C_{G}^{b}C_{e}^{G}]\delta R^{e}\\ & +A[C_{b}^{u}(C_{G}^{b}C_{e}^{G}P_{br}^{e}\times )-C_{b}^{u}(P_{bu}^{b}\times )]\psi_{U}\\ & =H_{\phi }\phi +H_{{\delta R}^{e}}\delta R^{e}+H_{\psi_{U}}\psi_{U} \end{array}$$

Finally, the USBL observation model of the integrated system can be given as:

(25) $$Z_{USBL}=\left[\begin{array}{c} \alpha_{S}-\alpha_{U}\\ \beta_{S}-\beta_{U}\\ R_{S}-R_{U} \end{array}\right]=\left[\begin{array}{c} {\delta \alpha }_{S}-{\delta \alpha }_{U}\\ {\delta \beta }_{S}-{\delta \beta }_{U}\\ {\delta R}_{S}-{\delta R}_{U} \end{array}\right]=H_{USBL}X+v_{USBL}$$

where $H_{USBL}=\left[\begin{array}{cccccc} H_{\phi } & 0_{3\times 3} & H_{{\delta R}^{e}} & 0_{3\times 9} & -H_{U} & H_{\psi_{U}} \end{array}\right]$.

## 4. Design of the Modified Filter Algorithm and Vector Fault Detection Method

### 4.1. The Modified Kalman Filter for Sparse Measurement Signals

The Kalman filter (KF) is a linear recursive filter algorithm based on the linear minimum variance. The KF filter runs in real time so it has numerous applications in navigation.

It is supposed that there are N measurements with different update frequencies involved in the KF, and their update periods are $T_{1}$, $T_{2}$, …, $T_{N}$. The KF update period $T_{K}$ is designed as the common multiple in the conventional algorithms, and it can be expressed as:

(26) $$T_{K}=n_{i}•T_{i}\;(i=1,2,\ldots ,N)$$

where $n_{i}$ is an integer that defines the update period. 

The employed integrated filter model is an approximate linear model and the KF is a linear algorithm. If the interval between two adjacent filter updates is too long, the filter may lose its precision especially for the high dynamic range. However, the output of the USBL is one kind of sparse measurement signals which will decrease the filter update frequency. Therefore, the conventional KF is not the optimum filter algorithm for the grid SINS/DVL/USBL integrated navigation. 

In this section, an MKF is employed to improve the update frequency of filter in this paper. The MKF update period $T_{K}$ is designed as the common divisor of measurements, and it can be expressed as:

(27) $$T_{i}=n_{i}•T_{K}\;(i=1,2,\ldots ,N)$$

where $n_{i}$ is an integer that defines the update period.

If the USBL output has not been updated, the filter works in the time update mode. In this case, the filter steps are expressed as:

(28) $${\hat{X}}_{k}=\Phi_{k,k-1}{\hat{X}}_{k-1}$$

(29) $$P_{k}=\Phi_{k,k-1}P_{k-1}\Phi_{k,k-1}^{T}+\Gamma_{k-1}Q_{k-1}\Gamma_{k-1}^{T}$$

When the USBL output has been updated, the filter works in the time and measurement update mode. In this case, the filter steps are expressed as:

(30) $${\hat{X}}_{k,k-1}=\Phi_{k,k-1}{\hat{X}}_{k-1}$$

(31) $$P_{k/k-1}=\Phi_{k,k-1}P_{k-1}\Phi_{k,k-1}^{T}+\Gamma_{k-1}Q_{k-1}\Gamma_{k-1}^{T}$$

(32) $$K_{k}=P_{k/k-1}H_{k}^{T}{\left(H_{k}P_{k/k-1}H_{k}^{T}+{\bar{R}}_{k}\right)}^{-1}$$

(33) $${\hat{X}}_{k}={\hat{X}}_{k/k-1}+K_{k}(Z_{k}-H_{k}{\hat{X}}_{k/k-1})$$

(34) $$P_{k}=\left(I-K_{k}H_{k}\right)P_{k/k-1}{\left(I-K_{k}H_{k}\right)}^{T}+K_{k}R_{k}K_{k}^{T}$$

The update frequency of the MKF will not be influenced by the uncertain and low update frequency of the USBL. The MKF can also be employed by other integrated navigation systems to effectively deal with the sparse measurement signal. 

### 4.2. The Vector Fault Detection Method for Filters

To improve the fault tolerance of the polar grid SINS/DVL/USBL integrated navigation system, the fault detection vector is designed and the vector fault detection method is proposed in this paper. With the vector, the fault detection method can take the measurements as objects to detect and isolate the fault, and simultaneously improve the utilization of effective measurements, which can also improve the integration performance. In this section, a vector fault detection method based on the residual $\chi^{2}$ method, i.e., the residual vector $\chi^{2}$ fault detection method, is proposed as an example to detect and locate the mutant fault measurement. 

Firstly, the measurements as the detection object are classified based on the independence of fault’s occurrence, and can be rewritten as $Z={\left[\begin{array}{cccc} z_{1} & z_{2} & \cdots & z_{n} \end{array}\right]}^{T}$, where $z_{i}(i=1,2,\cdots ,n)$ is the measurement group which may contain more than one measurement. In the same group, the fault probabilities of different measurements are dependent. Whereas, the fault probabilities of different groups are independent from each other. The fault detection model of $z_{i}(i=1,2,\cdots ,n)$ is then established as:

(35) $${\hat{z}}_{i_{k}}=H_{i_{k}}(\Phi_{k,k-1}{\hat{X}}_{k-1})$$

where $H_{i_{k}}(i=1,2,\cdots ,n)$ is part of the observation matrix corresponding with $z_{i_{k}}$, and the subscript k denotes the k^th^ time-step, and ${\hat{z}}_{i_{k}}$ is the estimation of $z_{i_{k}}$. Taking the grid SINS/DVL/USBL integration as an example, the measurements can be classified as $Z={\left[\begin{array}{ccc} z_{\mathrm{DVL}} & z_{\alpha \beta } & z_{R} \end{array}\right]}^{T}$. The fault probabilities of $z_{\mathrm{DVL}}$, $z_{\alpha \beta }$ and $z_{R}$ are independent, but the fault probabilities of α and β are dependent. The fault detection models of $z_{\mathrm{DVL}}$, $z_{\alpha \beta }$ and $z_{R}$ are established as:

(36) $$\left\{ \begin{array}{l} {\hat{z}}_{DVL_{k}}=H_{DVL_{k}}(F_{k,k-1}{\hat{X}}_{k-1})\\ {\hat{z}}_{\alpha \beta_{k}}=H_{\alpha \beta_{k}}(F_{k,k-1}{\hat{X}}_{k-1})\\ {\hat{z}}_{R_{k}}=H_{R_{k}}(F_{k,k-1}{\hat{X}}_{k-1}) \end{array}\right.$$

where ${\left[\begin{array}{ccc} H_{DVL_{k}} & H_{\alpha \beta_{k}} & H_{R_{k}} \end{array}\right]}^{T}=H_{k}$.

Then, the residual of the measurement estimation $r_{k}$ can be obtained as:

(37) $$r_{k}=\left[\begin{array}{c} r_{1_{k}}\\ r_{2_{k}}\\ \vdots \\ r_{n_{k}} \end{array}\right]=\left[\begin{array}{c} z_{1_{k}}-{\hat{z}}_{1_{k}}\\ z_{2_{k}}-{\hat{z}}_{2_{k}}\\ \vdots \\ z_{n_{k}}-{\hat{z}}_{n_{k}} \end{array}\right]$$

If no fault occurs, $r_{i_{k}}(i=1,2,\cdots ,n)$ can be considered as white noise which obeys normal distribution, i.e., $r_{i_{k}}\sim N(0,A_{i_{k}})(i=1,2,\cdots ,n)$. The residual $r_{i_{k}}(i=1,2,\cdots ,n)$ has zero mean and the variance $A_{i_{k}}(i=1,2,\cdots ,n)$ is:

(38) $$A_{i_{k}}=H_{i_{k}}P_{k/k-1}H_{i_{k}}^{T}+R_{i_{k}}$$

When the measurement fault occurs, the statistical characteristics of measurement noise will change, and the residual $r_{i_{k}}$ is no longer white noise, which means the statistical characteristics of $r_{i_{k}}$ will change and the mean of $r_{i_{k}}$ is no longer zero. Then the statistical characteristics change of $r_{i_{k}}$ can be used to detect the measurement fault.

The fault detection vector is designed as:

(39) $$\Gamma =\left[\begin{array}{cccc} r_{1}^{T} & r_{2}^{T} & \cdots & r_{n}^{T} \end{array}\right]\left[\begin{array}{cccc} A_{1}^{-1} & 0 & \cdots & 0\\ 0 & A_{2}^{-1} & \cdots & 0\\ \vdots & \vdots & \ddots & \vdots \\ 0 & 0 & \cdots & A_{n}^{-1} \end{array}\right]\left[\begin{array}{cccc} r_{1} & 0 & \cdots & 0\\ 0 & r_{2} & \cdots & 0\\ \vdots & \vdots & \ddots & \vdots \\ 0 & 0 & \cdots & r_{n} \end{array}\right]=\left[\begin{array}{cccc} \gamma_{1} & \gamma_{2} & \cdots & \gamma_{n} \end{array}\right]$$

where Γ is the fault detection vector, and $\gamma_{i}(i=1,2,\cdots ,n)$ is the fault detection value of $z_{i}$. $\gamma_{i}$ obeys the $\chi^{2}$ distribution and its degree of freedom is m, i.e., $r_{i}\sim \chi^{2}(m)$. m is the dimension of measurement group $z_{i}$.

A detection flag vector $F=\left[\begin{array}{cccc} f_{1} & f_{2} & \cdots & f_{n} \end{array}\right]$ is defined, and the fault detection criteria is:

(40) $$f_{i}=\left\{ \begin{array}{l} 1;\;\gamma_{i}>T_{D_{i}},\;some\;faults\;occur\\ 0;\;\gamma_{i}\leq T_{D_{i}},\;no\;faults\;occur \end{array}\right.\;(i=1,2,\cdots ,n)$$

where $T_{D}$ is the threshold determined according to the false alarm rate. 

According to the detection flag vector F, if F is a zero vector, all the measurements perform well. However, if $f_{i}=1$, the fault occurs in the measurement group $z_{i}$ and the filter model should be reconstructed to isolate the measurement $z_{i}$.

**Figure 4:**
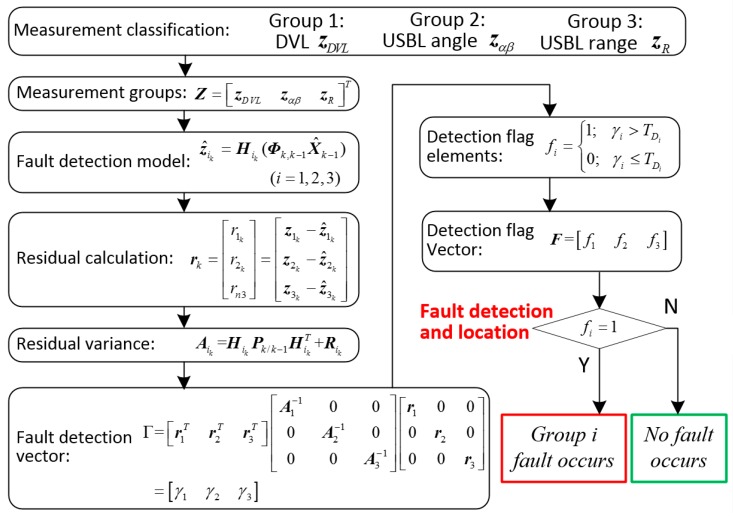
The flow diagram of the vector fault detection method.

Taking the grid SINS/DVL/USBL integration as an example, Figure 4 shows the flow diagram of the vector fault detection method for the integrated navigation system.

By designing and employing the detection vector, the vector detection method can locate the fault measurement instead of the fault filter, and fault measurement will be isolated by the reconstruction of observation matrix but the effective measurements will be kept. Therefore, the vector detection method can also be employed by the centralized filter. Except for the residual $\chi^{2}$ fault detection method, some other effective traditional detection methods can also employ a proper detection vector to improve the accuracy of location fault and meanwhile the performance of integrated navigation.

## 5. Experiment Results and Discussions

To validate the performance of the proposed grid SINS/DVL/USBL integrated algorithm, the simulations and experiments are conducted in this section. Besides, the sea state and vehicle motion status are considered. The sea state is set as moderate condition, and the motion status includes static, motion with constant velocity, motion with constant acceleration, diving and turning a corner. The main inertial measurement unit (IMU) and motion parameters of the simulation experiment are described as follows.

Firstly, the gyroscopes drifts are set as 0.05 degrees per hour and the random errors of gyroscopes are set up as zero-mean Gaussian white noises. The accelerometer biases are set as 6 × 10^−5^ g and the random errors of accelerometers are set up as zero-mean Gaussian white noises.

Secondly, the attitude of the vehicle is set as a sine function to simulate the influence of the moderate sea state. The amplitude and period (denoted as amplitude/period) of pitch angle, roll angle and yaw angle are set as 2°/7 s, 3°/9 s and 1°/8 s, respectively.

Thirdly, the acceleration of the vehicle is set as 0.2 m/s^2^ and the vehicle has the maximum speed 5 m/s. The latitude and longitude of the initial position *P* is set as (75° N, 126° E). To facilitate understanding, the position errors of navigation algorithms are expressed as the position errors along the longitude, latitude and altitude direction respectively, and the unit of position errors is meter.

Finally, the DVL output frequency is 1 Hz, and the USBL output frequency is 0.2 Hz. The DVL velocity error is less than 0.3 m/s. The USBL range measurement error is less than 20 m, and the angle error is less than 1.5°.

### 5.1. Simulation Results and Discussions

In this section, the simulation experiments are conducted to assess the performance of the proposed grid SINS/DVL/USBL integrated navigation algorithm, including the integrated navigation algorithm and the vector fault detection method. 

#### 5.1.1. Comparisons of the Integrated Navigation Algorithms

There are three polar integrated navigation algorithms discussed in this section, i.e., the traditional grid SINS/DVL integrated algorithm, the traditional grid SINS/DVL/USBL integrated algorithm 1 and the proposed grid SINS/DVL/USBL integrated algorithm 2. The traditional grid SINS/DVL/USBL 1 employs the absolute position as measurements, and the proposed grid SINS/DVL/USBL 2 employs the relative position as measurements. All the three integrated navigation algorithms choose the MKF as the filter algorithm.

**Figure 5:**
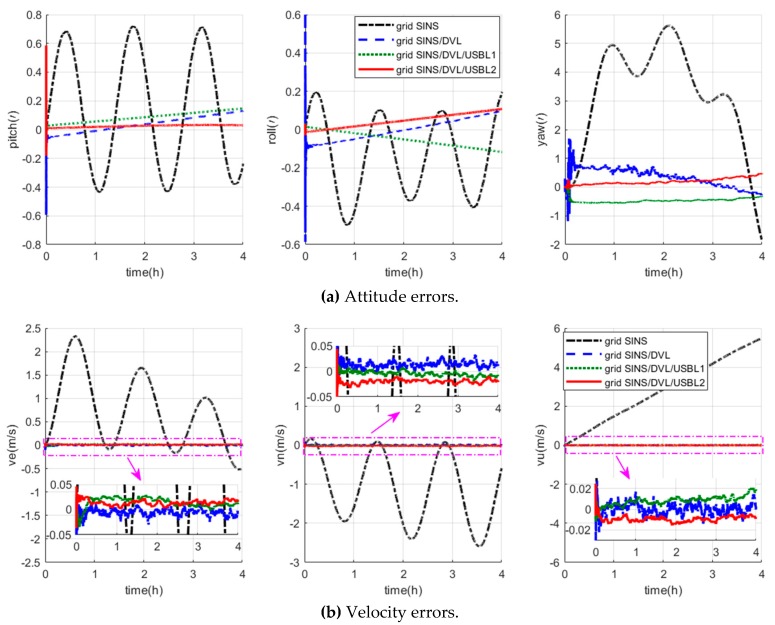

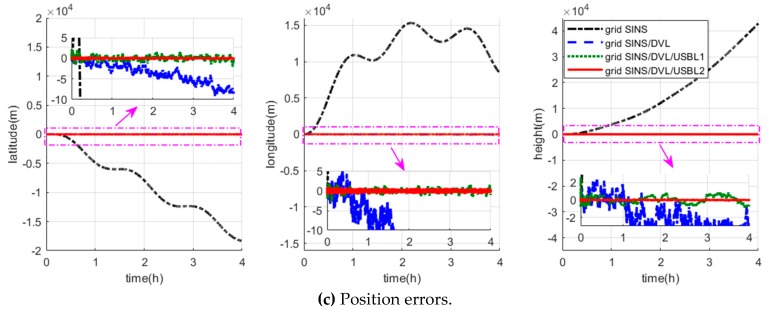
Description of the navigation errors of four algorithms.

The navigation errors of the grid SINS and three integrated algorithms are depicted in Figure 5. Besides, Table 1 shows the position error statistics of the grid SINS/DVL/USBL integrated navigation algorithms with different USBL position measurements.

As shown in Figure 5, the grid frame can effectively restrain the navigation error amplification, and all the three integrated navigation algorithms can restrain the grid SINS errors effectively. As shown in Figure 5a,b, three integrated algorithms have almost the same attitude and velocity accuracy. However, the position errors of the grid SINS/DVL integrated navigation algorithm, shown as the blue curves in Figure 5c, still accumulate with time, which cannot maintain the necessary of high-precision polar navigation, and the grid SINS/DVL/USBL integrated navigation algorithms have higher position accuracy than the grid SINS/DVL algorithm. As shown in Figure 5c and Table 1, the position errors of both two grid SINS/DVL/USBL algorithms do not accumulate with time, and the proposed grid SINS/DVL/USBL algorithm (algorithm2) has higher accuracy than the traditional SINS/DVL/USBL algorithm (algorithm1). Compared to the traditional algorithm1, the latitude, longitude and height accuracies of the proposed algorithm2 are improved 88.44%, 57.34%, 41.41% (maximum error), and 79.43%, 55.83%, 87.56% (RMS), respectively. Therefore, compared to the traditional integrated algorithms, the proposed grid SINS/DVL/USBL integrated algorithm with relative position measurements can ensure the safety operation of ocean space vehicles in polar regions with high navigation accuracy.

#### 5.1.2. Simulation Experiments of the Vector Fault Detection Method

**Figure 6:**
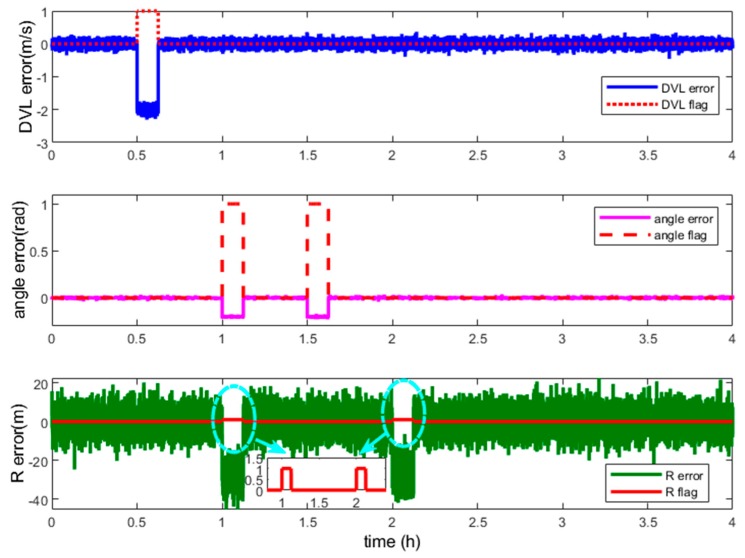
Description of the measurement fault and detection flags.

In this section, the performance of the vector fault detection method and the fault-tolerant grid SINS/DVL/USBL integration are discussed. The centralized MKF is the filter algorithm and the residual vector $\chi^{2}$ fault detection method is employed for the centralized filter. Some possible mutant fault measurements are involved in this experiment. The starting and termination times of the mutant measurement fault are shown in Table 2 and the measurement errors and the fault detection flags are shown in Figure 6.

As description in the Section 4.2, the measurements are classified as three measurement groups according to the independence of fault’s occurrence, i.e., the range *R* from the USBL, the direction angles ${\left[\begin{array}{cc} \alpha & \beta \end{array}\right]}^{T}$ from the USBL and the velocity from the DVL. The R flag is the fault detection flag of the USBL measurement *R*. The angle flag is the fault detection flag of the USBL measurement ${\left[\begin{array}{cc} \alpha & \beta \end{array}\right]}^{T}$. The DVL flag is the fault detection flag of the DVL measurement. If no fault occurs, the corresponding flag is 0. If some fault occurs, the corresponding flag is 1. The detection flag vector contains three elements, i.e., the DVL flag, angle flag and R flag. The fault measurement can be identified by its flag in the vector. The measurement errors and corresponding flags are shown in Figure 6.

As the flag lines showing in Figure 6, the DVL fault during 1800 s to 2250 s and the USBL fault during 3600 s to 4050 s both can be detected and located by the detection vector. During the time 5400 s to 5850 s β and α contain mutant fault but R works normally. So the angle flag becomes 1 and the R flag is still 0, and only the angle measurements, i.e., α and β, are isolated to reconstruct the filter. Then, during the time 7200 s to 7650 s, *R* contains mutant fault but α and β work normally. So the R flag becomes 1 and the angle flag is still 0, and only the R measurement is isolated to reconstruct the filter. Owing to the detection vector, the fault measurements of the centralized filter can be detected and located effectively. Especially, when the measurement device contains two kinds of navigation information, the fault occurrence of which is mutually independent, such as the angle $\left[\begin{array}{cc} \alpha & \beta \end{array}\right]$ and R from the USBL in this experiment, the fault measurements can also be located. The vector detection method successfully takes the measurement as object to locate the fault, so the fault location has more accuracy than the traditional scalar method and the utilization of effective measurements has been improved.

**Figure 7:**
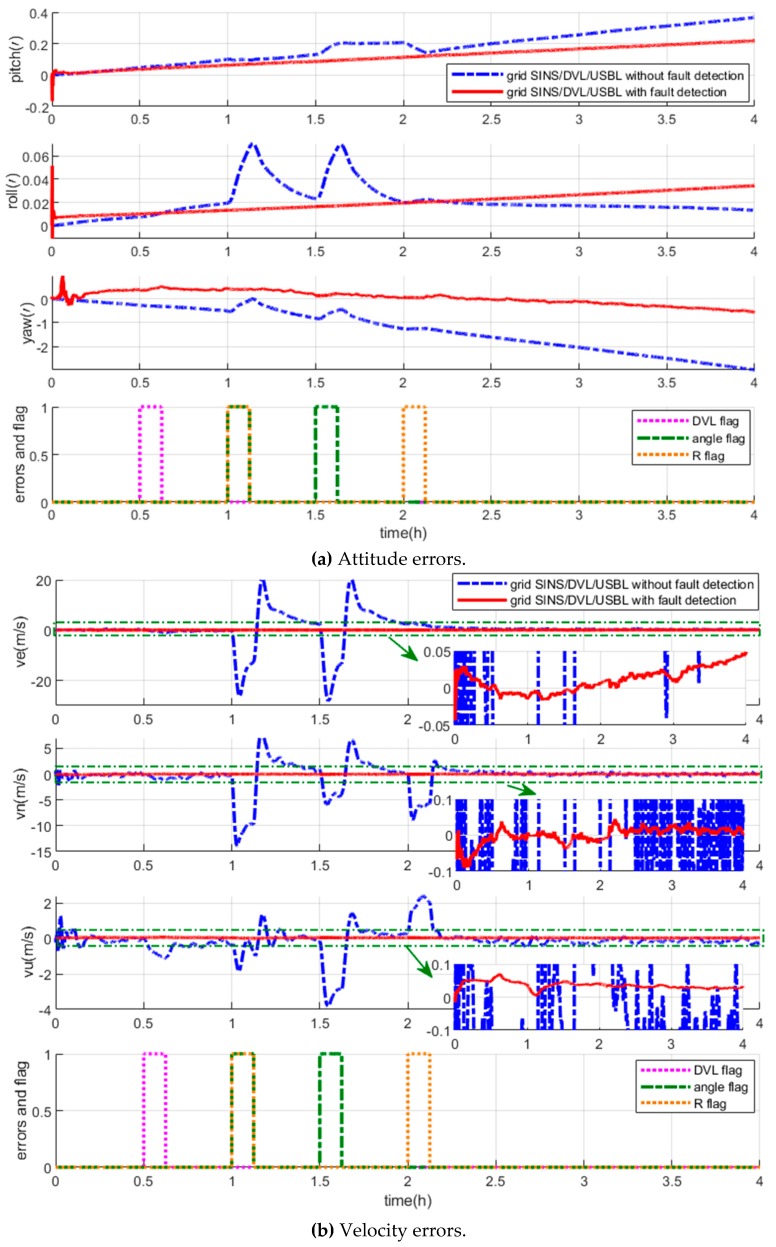

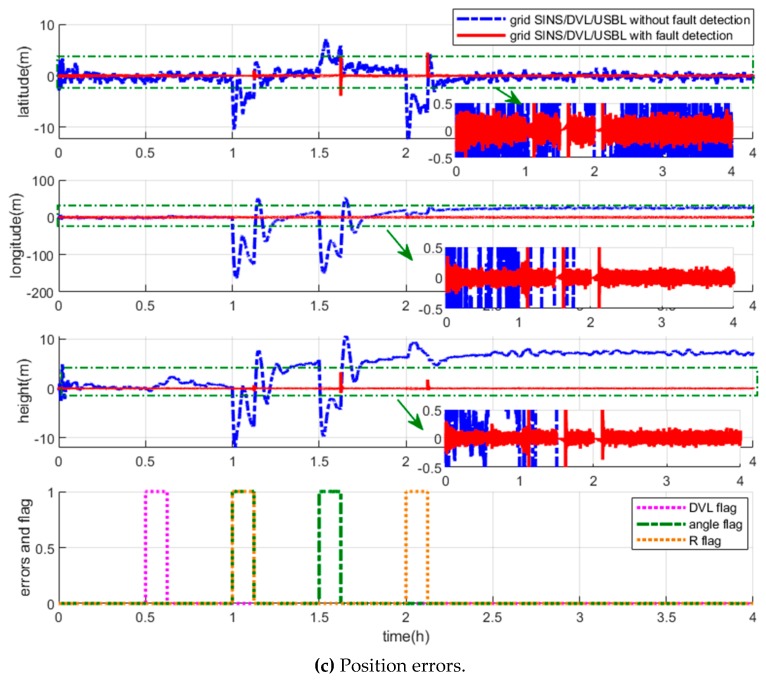
Description of the grid SINS/DVL/USBL integrated navigation errors with and without the fault detection.

The navigation errors in this experiment are shown in Figure 7. The blue curves indicate the errors of the grid SINS/DVL/USBL integrated algorithm without the fault detection, and the red curves indicate the errors of the grid SINS/DVL/USBL integrated algorithm with the vector fault detection method.

As shown by the blue curves in Figure 7, when the measurements fault occurs, the integrated navigation algorithm without the fault detection loses its accuracy. However, with the fault detection, the fault can be detected and located by the fault detection vector, and then the filter is reconstructed to isolate the fault measurement immediately. In this case, as shown by the red curves in Figure 7, the integrated navigation can maintain high accuracy because the reconstructed filter keeps updating uninterruptedly and accurately. On the other hand, the uninterruped filter ensures the uninterrupted fault detection. Therefore, when the measurements return to normal, the recovery is detected, and the isolated measurements are introduced into the integrated navigation filter again by another reconstruction without an obvious convergence time. 

As a whole, the proposed vector fault detection method chooses the measurement as objects to locate the fault by employing the detection vector, so it also can detect and locate the fault measurement effectively when employed by the centralized filter. With the help of the vector fault detection method, the grid SINS/DVL/USBL integrated navigation algorithm can improve the fault tolerance and maintain the accuracy when some measurement mutant fault occurs.

### 5.2. Semi-Physical Experiment Results and Discussions

In this section, the semi-physical experiments are conducted to further validate the performance of the proposed algorithm due to the geographic restriction of the polar experiment. The semi-physical experiments are designed based on two principal factors which mainly affect the performance of the standard polar and ocean navigation strategies, i.e., the amplification of navigation errors caused by the meridian convergence and the navigation precision mainly influenced by the IMU measurement errors. Therefore, the semi-physical experiment, which can simulate the polar meridian convergence and simultaneously contain the actual IMU output errors, will be more realistic to verify the performance of the proposed navigation algorithm. 

Moreover, compared to the simulation experiments, more standard navigation strategy and experiment condition are involved in the semi-physical experiment [27]. The GNSS signals are employed as measurements to give more comparative common navigation strategies and the vehicle trajectory is also designed to include both the surface environment where the GNSS can provide position coordinates and also the underwater environment where the GNSS signals are lost. Then the simultaneous faults of the DVL and USBL are involved to verify the performance of fault detection method.

The semi-physical experiment contains three parts, i.e., the extraction of sensor errors and generation of the semi-physical experiment data, the performance validation of the integrated algorithms, and the performance validation of the fault detection method, which will be discussed in the following subsections.

#### 5.2.1. Extraction of Sensor Errors and Generation of the Semi-Physical Experiment Data

In this subsection, a set of polar semi-physical experiment data that contain the actual IMU output errors and ideal IMU outputs is generated and will be employed to verify the performance of the algorithm. In this experiment, the ideal IMU outputs in polar regions can be generated by the trajectory generator, and the key point to get the semi-physical experiment data is to get the actual IMU output errors.

**Figure 8:**
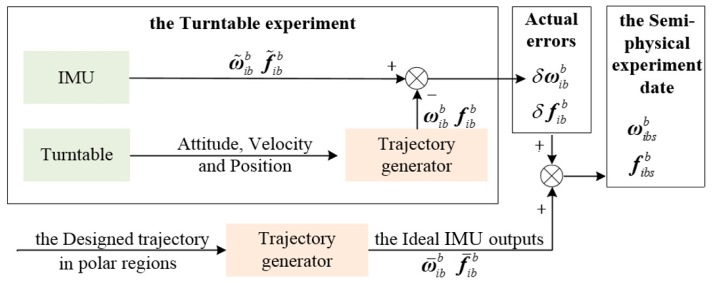
Description of signal flow to get the semi-physical experiment data.

The signal flow to get the actual IMU output errors, i.e., $\delta \omega_{ib}^{b}$ and $\delta f_{ib}^{b}$, and the semi-physical experiment data, i.e., $\omega_{ibs}^{b}$ and $f_{ibs}^{b}$, is shown as Figure 8.

**Figure 9:**
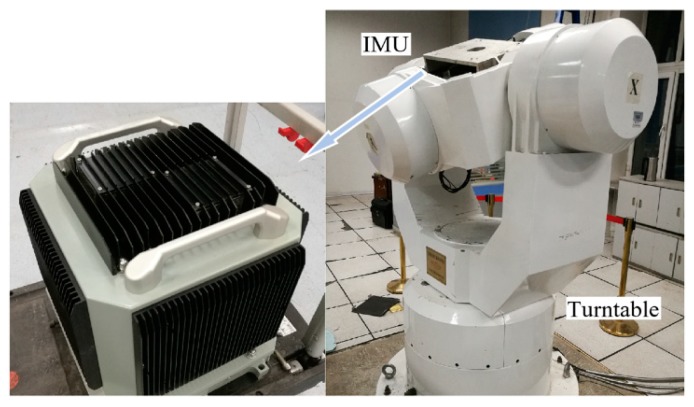
Description of the equipment of the turntable experiment.

In the turntable experiment, an IMU is installed on a high-precision three-axis turntable as shown in Figure 9. The main parameters of the three-axis turntable and IMU are shown in Table 3 and Table 4. 

During the turntable experiment, the IMU output, i.e., ${\tilde{\omega }}_{ib}^{b}$ and ${\tilde{f}}_{ib}^{b}$, and the turntable movement data are collected. Then, a trajectory generator is employed, which can generate the ideal reference of IMU output, i.e., $\omega_{ib}^{b}$ and $f_{ib}^{b}$, when the vehicle’s movement is input or designed. As shown in Figure 8, the movement data of the turntable, i.e., the attitude, velocity, and position, are inputted into the trajectory generator, and the reference IMU output data, i.e., $\omega_{ib}^{b}$ and $f_{ib}^{b}$, are generated. 

Then the actual IMU output errors are obtained as:

(41) $$\left\{ \begin{array}{l} \delta \omega_{ib}^{b}={\tilde{\omega }}_{ib}^{b}-\omega_{ib}^{b}\\ \delta f_{ib}^{b}={\tilde{f}}_{ib}^{b}-f_{ib}^{b} \end{array}\right.$$

The $\delta \omega_{ib}^{b}$ and $\delta f_{ib}^{b}$ are the actual IMU output errors gained from the above turntable experiment.

Meanwhile, the trajectory generator is designed to simulate the polar region geographic environment which leads to the meridian convergence. The ocean space vehicle trajectory in polar regions and the corresponding ideal IMU outputs, i.e., ${\bar{\omega }}_{ib}^{b}$ and ${\bar{f}}_{ib}^{b}$, are generated by the polar trajectory generator. 

Therefore, by adding the actual IMU errors, i.e., $\delta \omega_{ib}^{b}$ and $\delta f_{ib}^{b}$, to the ideal outputs, i.e., ${\bar{\omega }}_{ib}^{b}$ and ${\bar{f}}_{ib}^{b}$, a set of semi-physical experiment sensor output data, i.e., the $\omega_{ibs}^{b}$ and $f_{ibs}^{b}$, can be obtained. Then the semi-physical sensor output data are employed to conduct the following performance validation experiments.

#### 5.2.2. Semi-Physical Experiments of the Integrated Navigation Algorithms

The standard navigation strategies for ocean vehicles include the SINS/DVL integration, SINS/DVL/GNSS integration and SINS/DVL/USBL integration, in which the DVL provides the velocity and the GNSS and USBL provide the absolute position. The grid SINS/DVL/USBL integrated navigation algorithm proposed in this paper employs the DVL to provide velocity and the USBL to provide the relative position as measurements. 

There are four integrated navigation algorithms discussed in this section including the standard strategies and the proposed novel strategy:

Algorithm 1: the standard grid SINS/DVL integrated navigation algorithm;

Algorithm 2: the standard grid SINS/DVL/GNSS (absolute position measurement) integrated navigation algorithm;

Algorithm 3: the standard grid SINS/DVL/USBL1 (absolute position measurement) integrated navigation algorithm;

Algorithm 4: the proposed grid SINS/DVL/USBL2 (relative position measurement) integrated navigation algorithm.

**Figure 10:**
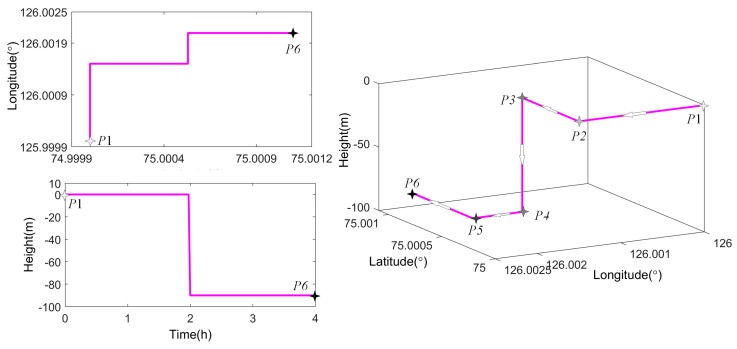
Description of the designed vehicle trajectory.

As shown in Figure 10, the first three waypoints of the vehicle trajectory (P1–P3) are on the surface where the GNSS can provide position coordinates. Then the vehicle descends to depths of about 90 m where the GNSS signals are lost and the grid SINS/DVL/GNSS strategy is switched to the grid SINS/DVL strategy.

The corresponding semi-physical sensor output data obtained in Section 5.2.1 is employed in this experiment. Besides, all the integrated algorithms choose the MKF as the filter algorithm. 

**Figure 11:**
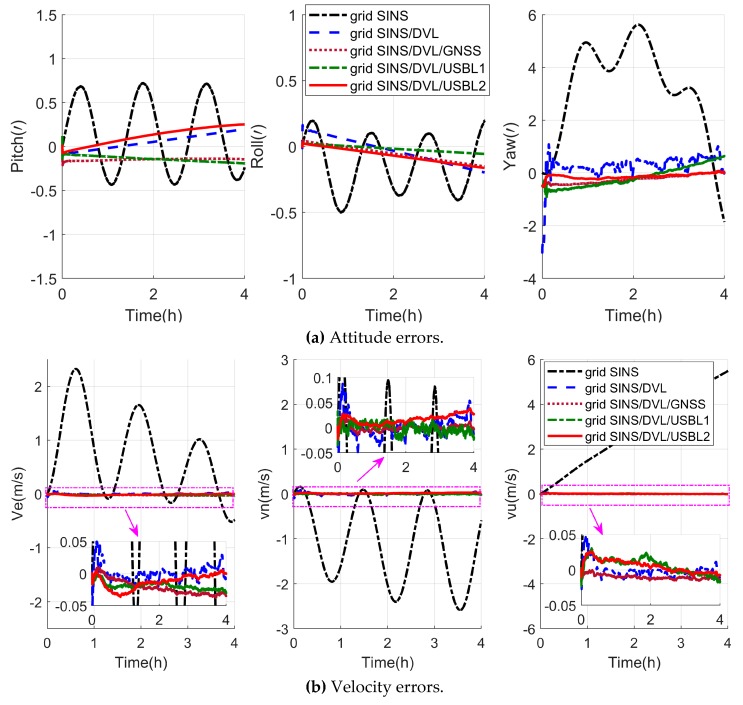

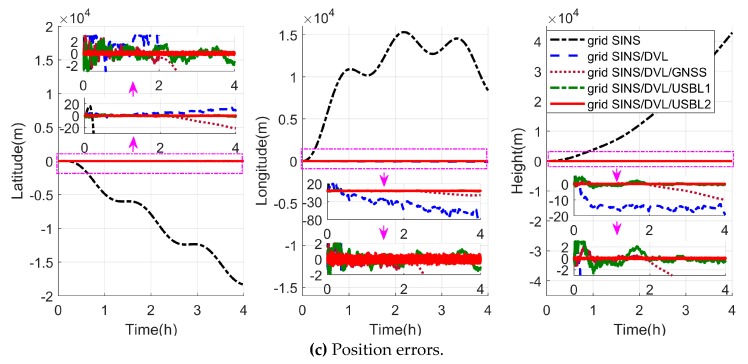
Description of the navigation errors in the semi-physical experiment.

The navigation errors of different algorithms in the semi-physical experiment are depicted in Figure 11. Besides, the position errors of grid SINS/DVL/USBL algorithms with different USBL position measurements are shown in Table 5.

**Figure 12:**
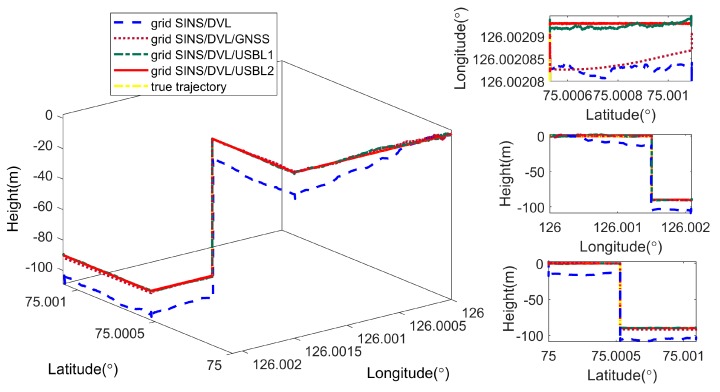
Position comparison between four integrated navigation strategies.

As shown in Figure 11, the proposed navigation algorithm can conquer the error amplification caused by the meridian convergence because of the grid navigation frame. As shown by the black curves, the grid SINS output still contains the periodic oscillation error and accumulated error, and the integration technique can restrain the grid SINS errors effectively with the help of velocity or position measurements from the DVL, GNSS and USBL. Besides, firstly the grid SINS/DVL, grid SINS/DVL/GNSS and the grid SINS/DVL/USBL integrated algorithm have nearly the same accuracy of attitude and velocity, but the position errors of the grid SINS/DVL integrated algorithm still accumulate with time. Secondly, when the vehicle operates on the surface, the position errors of the grid SINS/DVL/GNSS and the grid SINS/DVL/USBL do not accumulate, but when the vehicle navigates under water, the GNSS signals are lost and position performance between two navigation strategies becomes larger. Figure 12 shows the position comparison between four integrated navigation strategies. According to Figure 11 and Figure 12 and Table 5, the position accuracy of the proposed grid SINS/DVL/USBL 2 is higher than that of the traditional grid SINS/DVL/USBL 1 and compared to algorithm1, the latitude, longitude and height accuracies of algorithm2 are improved 87.38%, 63.84%, 91.73% (maximum error), and 87.01%, 65.26%, 89.36% (RMS), respectively. The maximum errors of latitude, longitude and height are reduced from 2.9663 m to 0.3742 m, 2.8152 m to 1.0179 m, and 2.5741 m to 0.2127 m, respectively, which can maintain the position accuracy of ocean space vehicles in polar regions.

#### 5.2.3. Semi-Physical Experiments of the Vector Fault Detection Method 

The proposed vector fault detection method and the fault-tolerant grid SINS/DVL/USBL integrated algorithm are discussed in this section. To validate the performance of the vector fault detection method, some mutant constant errors are added into the measurements. Compared to the simulation experiments, the ocean space vehicle trajectory and the corresponding semi-physical sensor output data obtained in Section 5.2.1 are employed in this experiment. Meanwhile, the possible condition, when part of USBL measurements and DVL measurements malfunction simultaneously, is involve in this semi-physical experiment. The starting and termination times of the measurement fault are shown in Table 6.

**Figure 13:**
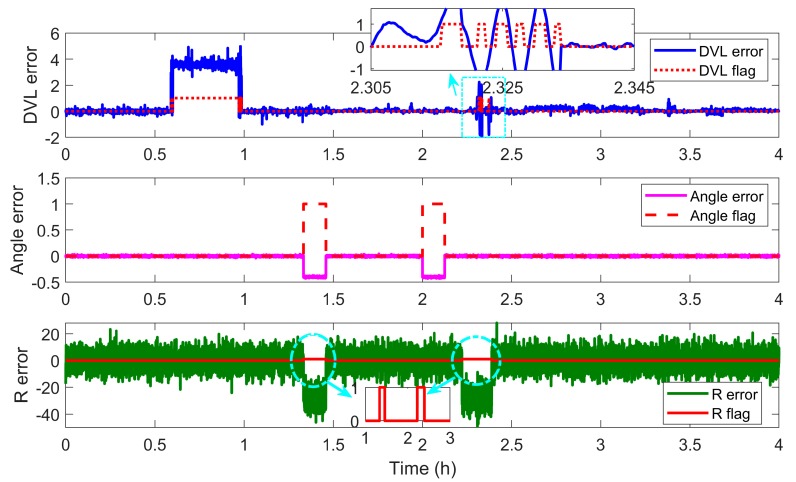
Description of the fault detection flags.

**Figure 14:**
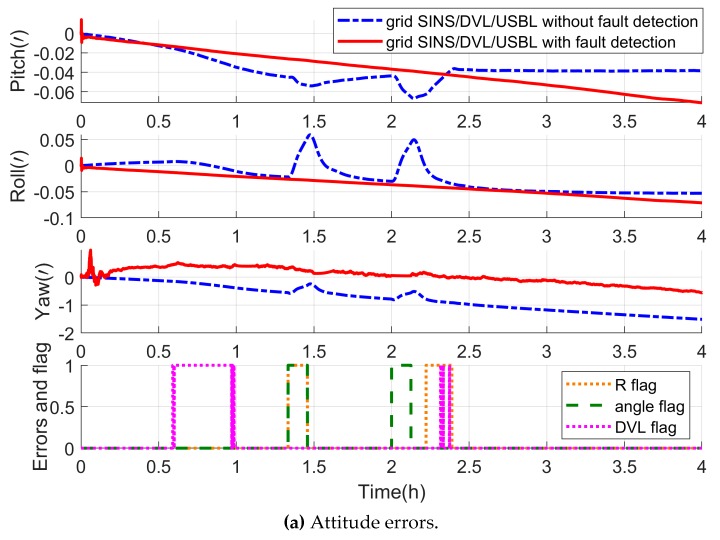

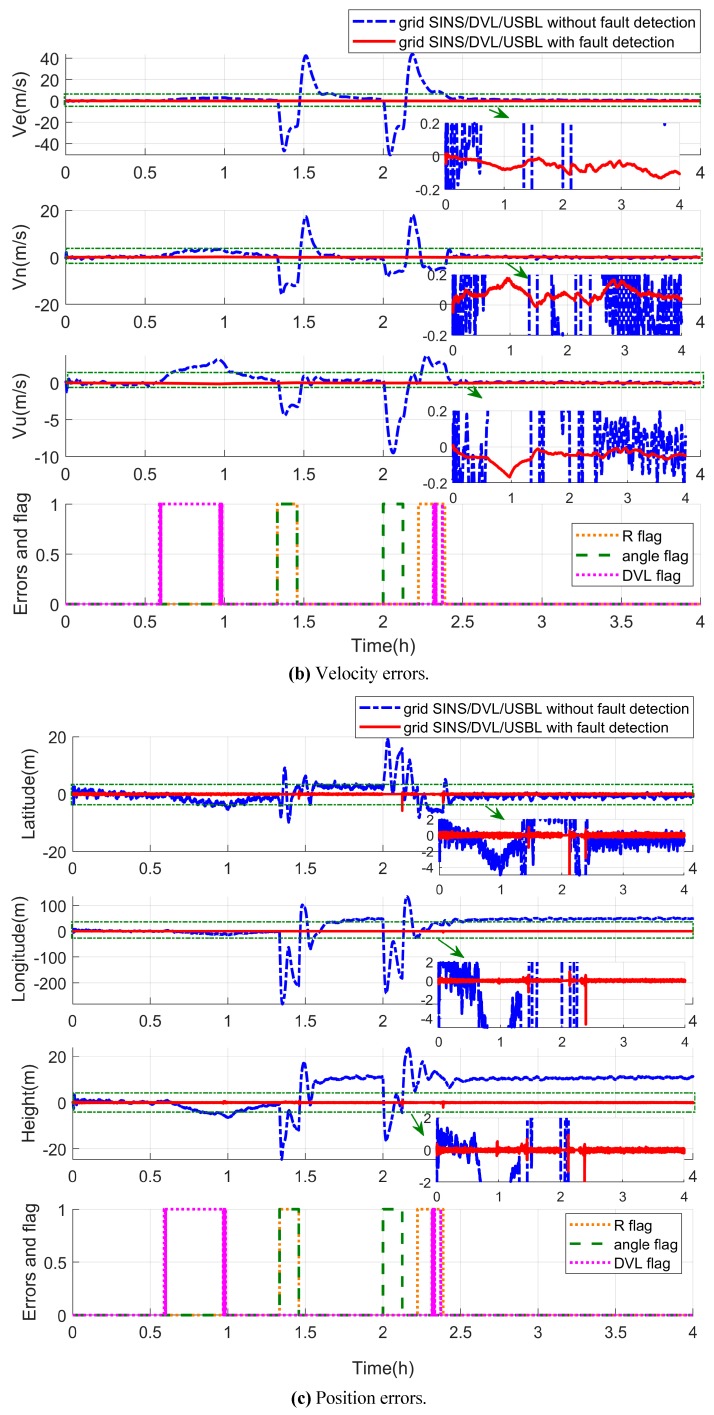
Description of the grid SINS/DVL/USBL integrated navigation errors with and without the fault detection.

The measurement errors and the fault detection flags are shown in Figure 13, and the navigation errors of the fault-tolerant grid SINS/DVL/USBL integrated algorithm are shown in Figure 14. 

As shown in Figure 13, different kinds of measurement fault, including the DVL fault, the USBL fault, the simultaneous DVL and USBL fault, can be detected and located by the proposed vector fault detection method.

Owing to the detection vector, the fault measurements can be isolated by the reconstruction of filter, and as the red lines showing in Figure 14, the integrated performance has not been influenced by the measurement fault. But as the blue curves show in Figure 14, when the measurement fault occurs, the grid SINS/DVL/USBL integrated navigation algorithm without the fault detection method loses its accuracy. 

Though the R measurements and angle measurements, i.e., α and β, which are both provided by the USBL, the fault of the two kinds of measurements are independent from each other. During the time 8261 to 8450 s, the integrated filter is challenged by both the velocity measurement fault from DVL and the R measurement fault from the USBL. Then the DVL and R measurements are located by the detection flag vector and isolated by the reconstruction. And meanwhile the filter is still operating with the α and β measurements to ensure the grid SINS errors were restrained uninterrupted during the short time. However, if the traditional scalar fault detection method is employed to isolate the simultaneous R and DVL fault, or the absolute position is employed as measurements, all the measurements would be isolated and the filter would be interrupted. As a result, the grid SINS errors would not be restrained by integration and the navigation accuracy would decrease sharply. Compared with the traditional scalar method, the vector method can improve the utilization of effective measurements and navigation performance. 

Hence, the proposed vector fault detection method can be employed by the centralized filter to detect and locate the fault measurements and improve the grid SINS/DVL/USBL integrated algorithm’s reliability and fault-tolerant to maintain the requirement of ocean space vehicles.

## 6. Conclusions

A fault-tolerant grid SINS/DVL/USBL integrated navigation algorithm based on the centralized filter and relative position information is proposed to improve the performance of ocean space navigation systems in polar regions. Firstly, the USBL and DVL output are employed as the measurements and the filter observation model of the USBL is proposed based on the relative position measurements to improve the USBL availability. Then, the grid SINS/DVL/USBL filter model is designed to improve the navigation accuracy. Moreover, considering of the sparse measurement from the USBL, the MKF is employed to improve the filter update frequency and estimate accuracy. Finally, the vector fault detection method, which takes the measurements as detection object instead of the traditional filter, is proposed to detect and isolate the measurement fault of centralized filter. Simulations and semi-physical experiment results show that the vector fault detection method can detect and isolate the measurement fault of centralized filter effectively, and together with the novel fault detection method, the grid SINS/DVL/USBL integrated algorithm can provide high-precision and high-reliable navigation information for ocean space vehicles to operate normally and safely in polar regions. Some important further developments are planned for the future. Firstly, if the conditions allow, a polar experiment is expected, and more kinds of external measurements will be employed to improve the integrated navigation accuracy and reliability.

## Figures and Tables

**Table 1:** The grid SINS/DVL/USBL integrated navigation position errors.

Parameters	USBL Measurement	Maximum	Improvement	RMS	Improvement
Latitude/(m)	Absolute position	2.0635	88.44%	0.6150	79.43%
	Relative position	0.2384		0.1265	
Longitude/(m)	Absolute position	2.7332	57.34%	0.5737	55.83%
	Relative position	1.1660		0.2534	
Height/(m)	Absolute position	0.9198	41.41%	0.4243	87.56%
	Relative position	0.5389		0.0528	

**Table 2:** The starting and termination time of measurement fault.

Fault Measurement	DVL	USBL	*α* and *β*	R
Starting time/(s)	1800	3600	5400	7200
Termination time/(s)	2250	4050	5850	7650

**Table 3:** Main parameters of the three-axis turntable.

	Outer Axis	Middle Axis	Inner Axis	Unit
Angular position accuracy	±3/1	±3/1.5	±3/1	arc-sec
Minimum angular rate	±0.001	±0.001	±0.001	°/s
Maximum angular rate	±180	±250	±400	°/s
Angular rate accuracy and stability	5 × 10^−5^	5 × 10^−5^	5 × 10^−5^	°/s
Angular rate resolution	0.0001	0.0001	0.0001	°/s

**Table 4:** Main parameters of the IMUs.

	Constant Bias	Random Bias
Gyroscope	<0.05 °/h	<0.05 °/h
Accelerometers	<7 × 10^−5^ g	<5 × 10^−5^ g

**Table 5:** The grid SINS/DVL/USBL integrated navigation position errors.

Parameters	USBL Measurement	Maximum	Improvement	RMS	Improvement
Latitude/(m)	Absolute position	2.9663	87.38%	0.9263	87.01%
	Relative position	0.3742		0.1203	
Longitude/(m)	Absolute position	2.8152	63.84%	0.7142	65.26%
	Relative position	1.0179		0.2481	
Height/(m)	Absolute position	2.5741	91.73%	0.4782	89.36%
	Relative position	0.2127		0.0509	

**Table 6:** The starting and termination times of measurement fault

Fault Measurement	DVL	USBL	α and β	R	DVL and R
Starting time/(s)	2100	4800	7200	8000	8261
Termination time/(s)	3550	5250	7650	8260	8450
